# Could painful temporomandibular disorders be nociplastic in nature? A critical review and new proposal

**DOI:** 10.2340/aos.v83.40586

**Published:** 2024-04-15

**Authors:** Peter Svensson

**Affiliations:** a Section for Orofacial Pain and Jaw Function, Department of Dentistry and Oral Health, Aarhus University, Aarhus C, Denmark; b Faculty of Dentistry, Malmø University, Malmö, Sweden; c Scandinavian Center for Orofacial Neurosciences, Aarhus University, Aarhus C, Denmark

**Keywords:** Temporomandibular disorder, orofacial pain mechanisms, quantitative sensory testing, trigeminal physiology

## Abstract

Classification of temporomandibular disorders (TMD) and, indeed, all types of orofacial pains has significantly progressed in the last decade based on international consensus work and operationalized clustering of signs and symptoms. A challenging gap nevertheless continues to exist in terms of understanding the underlying pain mechanisms and link to management. Recently, a novel mechanistic descriptor ‘nociplastic pain’ was introduced, and diagnostic algorithms and characteristic features were proposed. This narrative and critical review aim to discuss to what extent could painful TMD conditions fit into this category. Moreover, a number of less common types of orofacial pain could possibly also reflect nociplastic pain mechanisms. A model to differentiate TMD pain mechanisms is proposed, and the implications for management are discussed. The purpose of this review is to stimulate original and novel research into mechanisms of orofacial pain and hopefully thereby improve management of the individual patient.

## Classification of pain and orofacial pain

Pain is defined by the International Association for the Study of Pain (IASP) as ‘An unpleasant sensory and emotional experience associated with, or resembling that associated with, actual or potential tissue damage’ [1]. In order to provide a common language and tool for health care professionals IASP in collaboration with WHO and the International Classification of Diseases (ICD-11) recently developed a comprehensive classification of chronic pain [2, 3]. Chronic pain is now considered when the painful condition has lasted longer than 3 months and is separated into chronic primary pains where pain cannot be explained by another underlying disease or cause and where pain is the disease in its own right; and secondary pains where pain at least initially is caused by another disease or underlying cause and can be considered a symptom [3]. This distinction between pain as a disease versus pain as a symptom is crucial in the understanding of mechanisms and management. Regarding painful temporomandibular disorders (TMDs), they can also be classified as chronic primary pains when there is no underlying jaw muscle or joint pathology to explain the clinical presentation and as chronic secondary pains when, for example, rheumatoid arthritis is present with clinical manifestations in the temporomandibular joint (TMJ) [4]. The specific criteria for TMDs in the ICD-11 are adapted from the Diagnostic Criteria for TMD (DC/ TMD) [5] which was refined and improved from the Research Diagnostic Criteria for TMD (RDC/TMD) [6]. Thus, all the efforts to establish operationalized criteria and to test both reliability and validity for the DC/TMD are embedded in the ICD-11 criteria. The unique contribution of the original RDC/TMD was the inclusion of an axis I describing the clinical signs and symptoms in the jaw muscles, TMJ and adjacent tissues; and the axis II describing the psychosocial distress for example functional limitations, depression and somatization. This important feature of a pain classification system was introduced into the ICD-11 pain classification because of the direct implications for the management of the patients. Because orofacial pains and painful TMDs not all are chronic (> 3 pains) an additional and comprehensive classification of all types of orofacial pain was subsequently developed and published as the International Classification of Orofacial Pain (ICOP) [7]. In this comprehensive classification system all dentoalveolar, gingival, mucosal, salivary gland and bony types of pain as well as primary and secondary orofacial myofascial and TMJ pains, neuropathic pains and pains resembling chronic primary headaches in the orofacial region and finally idiopathic orofacial pain are described with specific criteria. A particular time-domain feature was introduced for the orofacial myofascial and TMJ types of pain as acute, chronic infrequent, chronic frequent and chronic highly frequent to better describe the ‘chronalgia’ of the painful condition.

In summary, important progress has occurred during the last decade in terms of classification of orofacial pains including painful TMDs but essentially the specific criteria rely on a systematic clustering of specific symptoms and clinical signs without addressing putative underlying pain mechanisms.

## Understanding pain mechanisms

Woolf et al. [8] in a milestone publication proposed to consider a mechanism-based classification of pain. Briefly, they highlighted at least four separate types of pain: (1) transient or nociceptive pain; (2) inflammatory pain; (3) neuropathic pain and (4) functional pain. This proposal was also adapted to orofacial pain [9, 10]. Transient or nociceptive pain is the normal physiological response of the somatosensory system when peripheral nociceptors are adequately stimulated by thermal, mechanical or chemical stimuli that do not cause overt damage to the tissue. Once the nociceptive stimulus is removed, the pain will disappear that is there is a strong link between the peripheral input and the output in terms of reported pain. Inflammatory types of pain are characterized by a tissue lesion and the inflammatory response leading to sensitization of the primary afferent nerve fibers. The neurobiology of this response is well characterized and pain will typically disappear when tissue healing has occurred. Neuropathic pain is also characterized by a lesion but involving the somatosensory system, that is, a lesion of the primary afferent (traumatic lesion of the peripheral nerve) or in the central nervous system (CNS); for example, stroke or neurodegenerative diseases changing dramatically the normal nociceptive function. Neuropathic pain therefore involves upregulation of ion channels and receptors both in the peripheral and CNS leading to peripheral and central sensitization and potentially also altered endogenous modulatory pathways. Neuropathic pain is considered an irreversible process with spontaneous pain and hypersensitivity to both non-painful and painful somatosensory stimuli (i.e. allodynia and hyperalgesia). Importantly, these changes in somatosensory sensitivity are confined to the neuroanatomically relevant region. Finally, Woolf et al. [8] suggested the term functional pain for those kind of pain conditions where no peripheral activation of nociceptors or inflammatory or neuropathic pathology could be identified leaving the curious observation of an unexplained upregulation of secondary or higher-order neurons in the CNS, that is an amplification of the otherwise normal peripheral input. The specific term ‘functional pain’ was meant to reflect a somatization or idiopathic type of pain with no known cause or obvious explanation but was often confused with ‘pain on function’ which is a common observation in many musculoskeletal types of pain including painful TMDs. In particular, for the functional type of pain, it has been difficult to explore specific pain mechanisms and criteria but recent efforts from the IASP led to the new proposal of nociplastic pain which may replace ‘functional pain’.

## Proposed criteria for nociplastic pain

The IASP working group proposed a specific definition and criteria for nociplastic pain (Tables 1 and 2) [11]. The key is that both nociceptive and neuropathic pains are ruled out as per their description (Table 1). An essential part of the nociplastic pain criteria is then the observation of hypersensitivity to somatosensory stimuli in the painful region (criterion 4) and hypersensitivity to other types of sensory stimuli, for example, taste, olfaction and sound (criterion 3). This is presumably related to the hyperexcitability of the CNS. The proposed criteria can lead to a ‘possible nociplastic pain’ or ‘probable nociplastic pain’ diagnosis (Table 2) but currently not to a ‘definite nociplastic pain’ diagnosis.

**Table 1:** IASP definitions of pain.

**Nociceptive pain:** Pain that arises from actual or threatened damage to non-neural tissue and is due to the activation of nociceptors.
**Neuropathic pain:** Pain caused by a lesion or disease of the somatosensory nervous system.
**Nociplastic pain:** Pain that arises from altered nociception despite no clear evidence of actual or threatened tissue damage causing the activation of peripheral nociceptors or evidence for disease or lesion of the somatosensory system causing the pain.

From [1]

**Table 2:** Proposed criteria for nociplastic pain.

1. The pain is: A. Chronic (> 3 months) B. Regional (rather than discrete) in distribution; C. There is no evidence that nociceptive pain (a) is present or (b) if present, is entirely responsible for the pain; and D. There is no evidence that neuropathic pain (a) is present or (b) if present, is entirely responsible for the pain
2. There is a history of pain hypersensitivity in the region of pain: Any one of the following: A. Sensitivity to touch B. Sensitivity to pressure C. Sensitivity to movement D. Sensitivity to heat or cold
3. Presence of comorbidities: Any one of the following: A. Increased sensitivity to sound and/or light and/or odors B. Sleep disturbance with frequent nocturnal awakenings D. Fatigue E. Cognitive problems such as difficulty to focus attention, memory disturbances, etc.
4. Evoked pain hypersensitivity phenomena can be elicited clinically in the region of pain: Any one of the following: A. Static mechanical allodynia B. Dynamic mechanical allodynia C. Heat or cold allodynia D. Painful after-sensations reported following the assessment of any of the above alternatives.

Possible nociplastic pain: 1 and 4; probable nociplastic pain: 1, 2, 3, and 4

From [11]

Also, the clinical features presumably related to nociplastic pain have been discussed in a recent Delphi publication [12]. There is consensus on many such features (see Table 3). Nevertheless, the unique criteria for nociceptive, neuropathic and nociplastic pain are still in need for refinement before clinical guidelines can be established. The important message is that a number of musculoskeletal pain conditions including painful TMDs but also other chronic orofacial pains could be nociplastic in nature and that this recognition could be important also from a management perspective.

**Table 3:** Top 10 proposed signatures of nociplastic pain.

1. Diffuse, widespread, or poorly localized distribution of pain
2. Generalized hypersensitivity
3. Multiple somatic symptoms (e.g. fatigue, memory difficulties, concentration difficulties, sleep disturbances, mood disturbances)
4. Varying distribution of pain
5. Presence of hypersensitivity to stimuli (e.g. pressure, temperatue, sound, odor, taste, light)
6. Generally not responsive to local anesthetics
7. Variability or no consistency in descriptors
8. Generally not responsive to surgery
9. Inconsistent, confusing and ambiguous responses and findings to clinical tests that vary over session
10. No findings from imaging of body regions of potential relevance to the pain experience

From [12]

It should be noted that the term nociplastic pain continues to trigger critical discussion and the underlying neurobiology remains elusive [13]. Nevertheless, it may be a useful and pragmatic approach to elaborate on the specific features for a better understanding of nociplastic pain in many chronic primary pain conditions including TMD pain.

## Indications that painful TMDs could be nociplastic pain

Temporomandibular disorders pain is typically chronic (> 3 months), described as a regional and often bilateral pain around the ear, the angle/body of the mandible and the temporal region which is diffuse and difficult to precisely locate [14]. This description is in complete agreement with the first criterion for nociplastic pain (Table 2). Also, a history of pain on jaw movement is a stereotypic observation in painful TMD cases [14] in line with the second criterion (Table 2). Indications of co-morbidities such as fatigue, poor sleep and cognitive problems as well as psychosocial distress, disability, non-specific physical symptoms and depression are other key features associated with TMD pain [14, 15]. It could be argued that the three first criteria mentioned in Table 2 are embedded in the axis I and II from the RDC/TMD and DC/TMD.

Somatosensory disturbances are a hallmark of nociplastic pain (Criterion 4, Table 2) and could clinically be interpreted as pain on palpation of the jaw muscles and/or TMJ which is required to establish a TMD pain diagnosis [5]. More standardized assessment of somatosensory function with the use Quantitative Sensory Test (QST) methods has, indeed, been used to describe somatosensory function in painful TMDs [14, 16, 17]. The consistent observation is that pressure pain thresholds are reduced in painful TMDs when compared to matched control individuals reflecting static mechanical allodynia. This observation is, however, not only true in the painful region (e.g. masseter and temporalis) but also in more remote and non-painful regions (e.g. the leg or arm) [18, 19]. Hypersensitivity to both mechanical and pressure stimulation of the TMJ region in patients with arthralgia has also been shown [20–22]. Hypersensitivity to both mechanical and thermal stimuli is also a consistent finding and replicated with full QST batteries in painful TMD patients [23, 24].

Mechanistically, central sensitization has been suggested to account for the alterations not only in mechanical pathways but also in thermal pathways extending well beyond the painful region in TMD pain patients; however, there is an ongoing debate about this use of ‘central sensitization’ in nociplastic pain. Central sensitization is the fundamental neural principle that after strong peripheral input the second-order neuron is sensitized with characteristic development of secondary mechanical hyperalgesia [25]. This phenomenon has recently been demonstrated using a high-frequency electrical stimulation model of the volar forearm and reveals an increased propensity for painful TMD patients to develop a larger secondary mechanical hyperalgesia area [26]. The point is being made that central sensitization is a specific neuronal mechanisms which in contrast the hypersensitivity to various somatosensory stimuli at different body locations may not necessarily represent. Indeed, also temporal summation mechanisms and endogenous pain modulatory control may play a significant role in the presumed pathophysiology of painful TMDs although the evidence is only modest according to a recent systematic review [27]. However, endogenous inhibitory control as represented by the conditioned pain modulation (CPM) phenomenon may be decreased in painful TMDs but is sensitive to type of conditioning stimulation and site of application [28]. There is also good evidence of increased after-sensations in TMD pain patients [29]. It seems clear that painful TMD is associated with one or more types of hypersensitivity to somatosensory stimuli as outlined in the criteria for nociplastic pain.

In fact, the discussion about TMD pain being a potential type of nociplastic pain is also relevant for other chronic primary orofacial pains, for example, persistent idiopathic dentoalveolar/facial pain, burning mouth syndrome/disorder because somatosensory abnormalities and most often hypersensitivity have been linked to these conditions [17, 30].

Finally, one study in TMD pain patients has further elaborated on the hypersensitivity to other sensory modalities and found good evidence in support of increased sensitivity to auditory stimuli when compared to healthy matched individuals [31]. This observation obviously needs further study with other sensory modalities.

In summary, there is evidence that painful TMD not only is associated with hypersensitivity to somatosensory stimuli in the painful region but also extending to other non-painful body areas. The specific neuronal mechanisms are unlikely to represent central sensitization as per the original and neurobiological definition but rather to a more g eneralized hypersensitivity to both somatosensory and other sensory stimuli reflected in an upregulation of the CNS processing. This points to painful TMDs as more centrally mediated chronic pain conditions rather than peripherally originated or maintained conditions. However, there is also evidence in favor of a peripheral pain component in painful TMDs so how can this controversy be reconciled?

## Other mechanisms in painful TMDs

First of all, it is important to understand that studies using, for example, microdialysis in painful TMD patients have identified noxious substances and inflammatory mediators for example like glutamate, 5-hydroxytryptamine, interleukin 6, 7, 8 and 13 in the jaw muscles [32–34]. Also studies in patients with degenerative changes in the TMJ have clearly illustrated inflammatory mediators in the synovial fluid [35, 36]. Interestingly, a study using state-of-the-art imaging with magnetic resonance, cone beam computer tomography and ultrasound identified a group of TMJ pain patients with no signs of degeneration and a group with one or more imaging signs of inflammation and showed significant differences in the QST profile [22]. Thus, patients with degenerative changes in the TMJ had significant hyperalgesia to TMJ pressure stimulation. It could be speculated that there are unique QST profiles of patients with a nociplastic pain versus patients with an inflammatory/nociceptive pain.

The peripheral tissues could also play a role in micro-trauma induced by bruxism [33]. The link between bruxism and TMD pain is still controversial because studies using self-reports and clinical examination of potential consequences of bruxism do support a link and in particular between awake bruxism and TMD pain [37, 38]. However, studies with polysomnographic recordings or electromyographic recordings of jaw muscle activity during sleep do not support this link but in contrast a reverse relationship: patients with more jaw muscle activity seem to have less risk of TMD pain [39–41]. There could be at least two reasons for this inconsistency: First, it seems that bruxism is more often associated with unpleasant, but non-painful muscle symptoms such as fatigue, stiffness, tension and stress [42,43]. In fact, the monumental Orofacial Pain: Prospective Evaluation and Risk Assessment (OPPERA) study identified non-specific orofacial complaints as the second-most important risk factor for first onset TMD pain [44]. This could point towards semantic issues related to ‘pain’ and ‘non-painful but unpleasant muscle symptoms’ in the understanding of bruxism and TMD pain. The second reason could be related to the suggestion that TMD pains are heterogeneous in nature (primary/secondary, different chronalgia), that is there are multiple subtypes of TMD pain in accordance with the current classification [3, 4, 7]. This assumption that TMD pain constitutes at least two but distinct pain mechanisms will now be discussed.

## Proposed conceptual model

**Figure 1:**
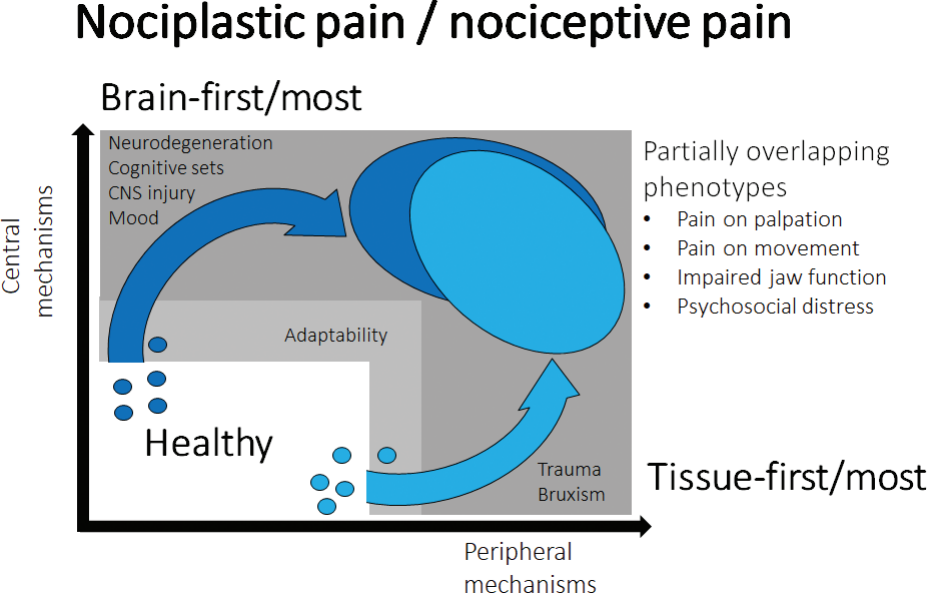
Proposed conceptual model of painful TMDs.

None of the existing TMD pain models discusses what kind of pain is involved in the complex interaction between multiple risk factors. For example, the gene x environment complex disease model highlights the interplay between neurobiological factors in terms of both tissue injury, peripheral and central sensitization and the multitude of psychological factors that can shape these interactions such as sleep, mood, psychosocial distress with an impact of genetic and epigenetic factors [45]. However, it is not clear what kind of TMD pain is the result of such factors and interactions. The present model is based on the assumption that clinical phenotypes may overlap for example the presentation of pain on jaw movement, pain on palpation, psychosocial distress and emotions but there may be two quite distinct mechanisms that will lead to these consequences. The obvious path is based on the traditional concept of tissue injury or overloading from macro-trauma and/or micro-trauma leading to peripheral sensitization and upstream sensitization of the second order and most likely higher order neurons in the CNS. In addition, there may be imbalance in the endogenous pain modulatory systems. It could be argued that this path is primarily peripherally driven (‘Tissue first – or tissue most’) but with an increasing amount of CNS involvement over time reflecting the dynamic changes of the nociceptive system (Figure 1). In contrast there could be a path originating within the CNS either due to detrimental impact of emotions and mood, initial degenerative changes or trauma to the CNS and due to an imbalance in descending pain regulatory pathways that is augmentation of descending facilitation which could lead to downstream sensitization. Most attention has been directed towards descending inhibitory pathways [46] and CPM effects [47] but in fact good evidence exist that there are serotonergic facilitatory pathways [48, 49]. This path could be labelled ‘brain first – or brain most’ and would therapeutically highlight interventions towards these domains (Figure 1). The latter is more consistent with the proposal of a nociplastic type of pain where the peripheral tissues appear normal and without nociceptive activity. The key in this model are the heterogeneous TMD pain mechanisms whereas the clinical phenotypes appear quite alike. This may be the simplest explanation for the continued controversies in terms of management strategies in TMD pain. Thus, the proposed model could have consequences in deciding management strategies.

## Implications for management

The most striking importance of the RDC/TMD was perhaps the inclusion of the two-axes system where management should take into consideration the degree of psychosocial distress and functional impairments for a better outcome and prognosis. Classical studies also clearly demonstrated the benefits of a comprehensive self-care program versus more ‘axis I’ approaches such as stabilization splits and over-the-counter types of medication [50]. As a continuation of this two-axes approach it could be suggested that a third-axis should be included which captured parts of the potential underlying mechanisms of the TMD pain. For example, there may specific brain signatures in patients with nociplastic pain so potentially more dorsolateral prefrontal activity would indicate a different pain mechanisms that more somatosensory-motor network activity [51]. Practical limitations may hinder such approaches in the clinic so other ‘proxies’ of nociplastic pain should be investigated and here simple QST within and outside the painful region may be helpful. Additionally, more refined CPM protocols could hold the potential not only to illustrate the endogenous inhibitory system but also facilitation when homotopic areas are being stimulated. Finally, the hypersensitivity to other sensory modalities could be exploited to better characterize nociplastic TMD pain. Neurocognitive interventions combined with neuromodulatory techniques for example repetitive transcranial magnetic stimulation could be alternative paths to more efficient management of subtypes of TMD pain. Likewise, it could be that peripherally targeted management with focus on overloading and potential inflammatory responses in the tissues would yield higher success rates if applied to more definitive nociceptive types of TMD pain. From the many decades of research into TMD pain it seems unlikely that ‘a new magic’ treatment will ever emerge but rather that new insights into which types of treatments should be applied to which types of TMD pain patients.

## Future research avenues

Hopefully, future research will test the proposed criteria for nociplastic pain and determine potential subgroups of painful TMD patients. QST appears to be a feasible way forward to help in this regard and to include standardized assessment of other sensory modalities as well. The true test of the concept of nociplastic pain will be if significant progress also will appear in the ability to predict efficacy in the individual patient management. The refinement of the proposed features of nociplastic pain is already in progress and new studies should test to what extent they apply to painful TMD conditions.

Animal research may also facilitate the understanding and some studies already claim to use nociplastic pain models. The challenge will be to understand the limitation of such models and the issues to extrapolate to the intact human being and going from nociception to pain. It should also be noted that the region of pain is a common criterion in the current classification but little is understood about the topography of painful TMDs in terms of representation of dermatomes and myotomes and to what extent spatial spread and referred pains complicate the localizability of pain.

## Conclusion and summary

The main message in this review is that it seems likely that, at least part, of the painful TMD patients could be related to nociplastic pain mechanisms but also that other painful TMD conditions could be nociceptive. The recognition of this heterogeneity of pain mechanisms in TMD pain is a fundamental step before management can be optimized and novel techniques and approaches developed and refined. Research is needed, and the current classification is a valuable prerequisite for pushing the field of orofacial pain forward in interdisciplinary and international collaborative efforts.

All healthy individuals will be exposed to a variety of risk factors which due to adaptability and resilience in the human body may not lead to longer-lasting detrimental effects. If an individual threshold is exceeded tissue damage or overloading may lead to activation and sensitization of peripheral primary afferent neurons and subsequent sensitization of second-order and higher-order neurons (upstream effects). The consequences will represent as the typical clinical phenotype of TMD pain (light blue). However, a similar clinical phenotype (dark blue) could also have originated through a brain-first/ most mechanism due to activation and sensitization of the CNS and involvement of descending facilitatory pathways (downstream effects). There may also be dynamic and time-dependent interactions between the proposed mechanisms but from a conceptual stand-point it may be important to recognize the fundamental difference between nociplastic and nociceptive pain.
